# Effects of solar tunnel drying zones and slice thickness on the drying characteristics of taro (*Colocasia esculenta* (L.) Schott) slice

**DOI:** 10.1002/fsn3.3175

**Published:** 2022-12-15

**Authors:** Ehtenesh Tilahun Molla, Tilahun A. Teka, Addisalem Hailu Taye

**Affiliations:** ^1^ Department of Postharvest Management, College of Agriculture and Veterinary Medicine Jimma University Jimma Ethiopia; ^2^ Department of Horticulture, Faculty of Agriculture Mizan Tepi University Mizan Tape Ethiopia

**Keywords:** *Colocasia esculenta*, drying zones, slice thickness, thermal properties

## Abstract

This study aims to investigate the effects of slice thicknesses (2, 4, and 6 mm) and solar tunnel drying zones (zone I, zone II, and zone III) on the drying characteristics and thermal properties of taro slices, which were dried using solar tunnel drying (STD). To assess the drying characteristics of taro slices, the data from the drying kinetics were fitted with five different models. The adequacy of fit for the proposed models was evaluated using the reduced chi‐square (*χ* ^2^), determination of coefficient (*R* ^2^), mean relative percent error (*P*), and root means square error (RMSE). The results showed that, among the five drying models, the drying characteristics of taro are better expressed by the logarithmic model. The thinnest slices dried in zone III had the highest diffusivity (6.57 × 10^–09^ m^2^/s), lowest specific heat capacity (1.761 kJ/kg °C), and maximum thermal conductivity (0.268 W/m °C). It was also dried within a short period of time (5.5 h). The findings of this study provide evidence that STD zones and slice thickness have significant impact on the drying characteristics of dried taro slices.

## Graphical abstract

The effects of different solar tunnel drying zones and slice thickness on the drying characteristics of taro slices were investigated. The data obtained were fitted with five selected models. Among the five drying models, the drying characteristics of taro are better described by the logarithmic model. The highest value of diffusivity was recorded for the thinnest slices dried in zone III.

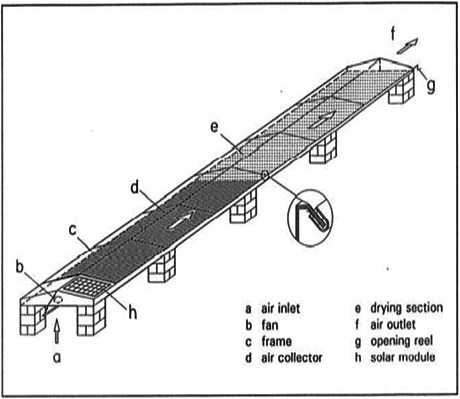

## 1 INTRODUCTION

Taro [*Colocasia esculenta* (L.) Schott] is among the most important root and tubers produced in subtropical and tropical parts of the world (Rashmi et al., 2018). It is commonly cultivated by resource‐constrained female farmers and smallholder farmers in Sub‐Saharan Africa (Otekunrin et al., 2021). Taro is the most important root and tuber crop next to cassava and yam produced and used for multipurpose in most African countries (Kaushal et al., 2015; Onyeka, 2014). In Ethiopia, taro ranks next to potato and sweet potato with regards to both coverage of area and production (Legesse & Bekele, 2021). Taro is a significant source of carbohydrates which serves as the cheapest dietary energy source. Besides, it is an important source of fiber and digestible starch while being low in protein and fat (Simsek & Nehir, 2015). The moisture content of fresh taro corms is high (63.6%–72.4%), and accounts two‐third of the total weight of the fresh crops (Huang et al., 2007). This is a major challenge for the taro roots’ storability since it causes a short shelf life. Previous studies showed that high moisture content creates favorable conditions for the growth of bacteria, mold, and yeast in root and tuber crops such as cassava (Chukwu & Abdullahi, 2015).

Drying is one of the well‐known and oldest methods of preservation of food. It reduces the deterioration of products and leads the perishable produce to be stable by removing a significant amount of moisture from the produce and retard microbiological and chemical activity of the produce (Hatamipour et al., 2007). In the food industry, hot‐air drying has been suggested as energy‐consuming process (Kocabiyik & Tezer, 2009). However, solar drying is one of the promising renewable sources of energy for the drying of food. It is ample, nonpollutant, and infinite in nature compared with fossil fuels shortage and its higher prices (Basunia & Abe, 2001).

The high solar intensity found in tropical area can be exploited as a source of solar energy to create solar tunnel drying. The high sun intensity in the area makes it economically viable to use solar tunnel drying to dry fruits and vegetables. Small scale businesses, rural areas, and areas with scarce of electric power can all benefit from using solar tunnel drying as an alternative form of drying (Sacilik, 2007), in longer solar tunnel drier (~16 m long), there is significant difference in the temperature and relative humidity of air at the intrance, the middle and exit part of the solar tunnel drier. Products exposed to high drying temperatures with low relative humidity (RH) dry faster than the products in the other drying zones. To the best of the authors' knowledge, there is a limited study about the effect of solar tunnel dryer zones and slice thickness on drying characteristics and thermal properties of taro yet in Ethiopia. Thus, the aim of this study was to investigate the effect of STD zones and slice thickness on the drying characteristics of taro slices.

## 2 MATERIALS AND METHODS

### 2.1 Sample preparation and collection

Fresh taro corms were obtained from the Jimma Agricultural Research Centre (JARC) of the Ethiopian Institute of Agricultural Research (EIAR), Jimma, Ethiopia. The washed taro corms were peeled and sliced in different thicknesses (2, 4, and 6 mm) by a food processor slicer (FP 700, China) using three different blades that can slice into 2, 4, and 6 mm slices and then dipped in 1% sodium chloride (NaCl) solution for 30 min. The soaked corm slices were dried using each of the drying zones (zone I zone II, and zone III) using an STD located at JUCAVM, Jimma University.

### 2.2 Description of STD

**FIGURE 1:**
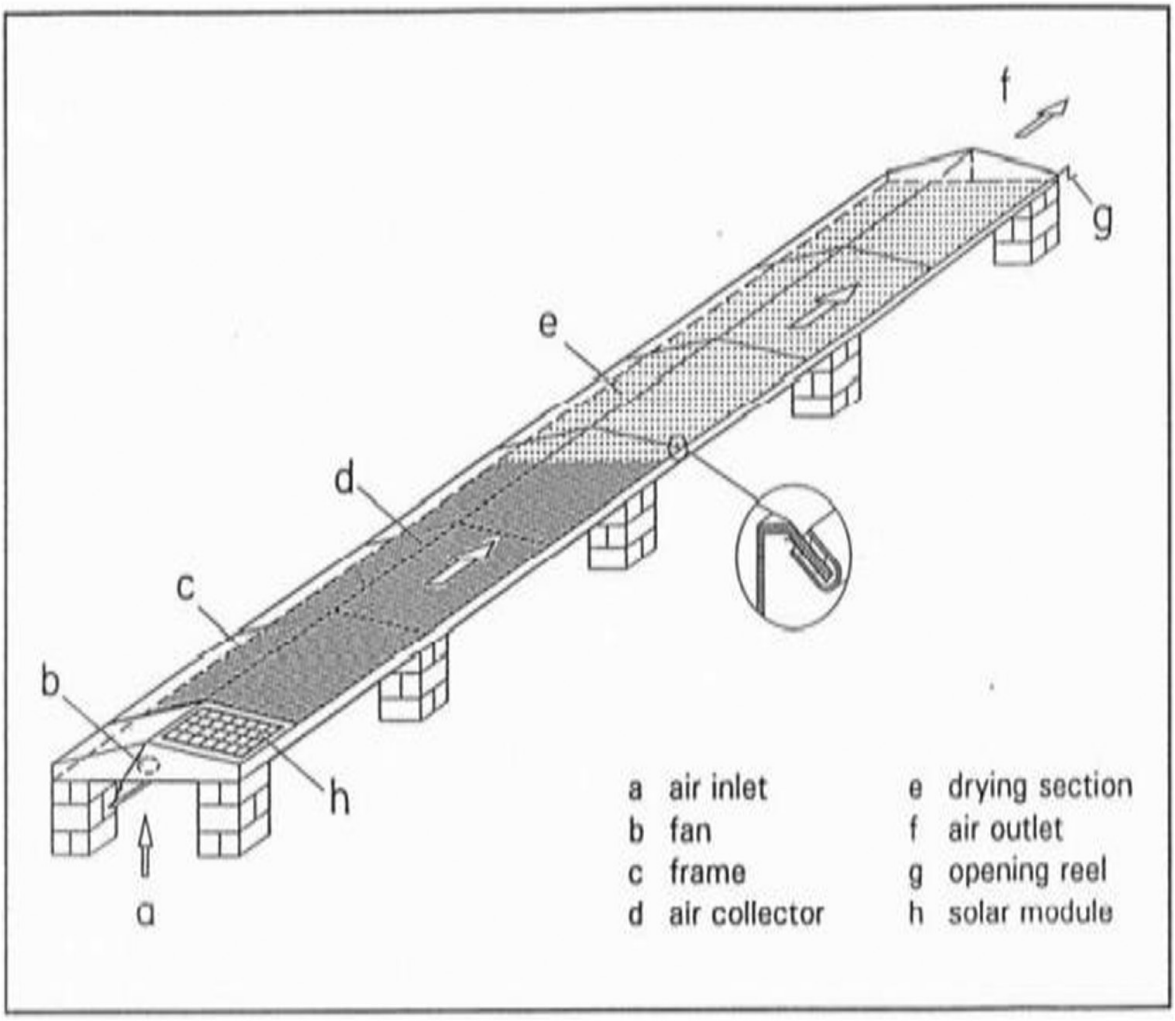
Diagram of solar tunnel dryer.

The schematic diagram of STD is presented in Figure 1. The solar STD has a length of 24 m and a width of 2 m. The solar collector is 8 m long and the remaining 16 m of the dryer is a drying zone. A solar panel was fixed on one side of the solar tunnel dryer to absorb the sun's rays as a source of energy generation, which is used as a supply for driving a fan. The air is drawn through the dryer by a fan. It is heated as it passes through the collector. During the preliminary test, data loggers at the inlet, middle, and outlet of solar tunnel drier were placed to read the temperature and relative humidity of the air. The result shows the presence of significant variation in the value of temperature and relative humidity. The total drying zone of 16 m has been subdivided into three zones each 5.33 m long. Since the variation in temperature and relative humidity are experimental factors for drying process, the STD zones were considered as a factor in this experiment. The inlet, middle, and outlet parts of STD are used as zone I, zone II, and zoneIII, respectively. The temperature and relative humidity of the ambient air and the drying zones were recorded by using data acquisition devices (Testo, model 184 H1). The data acquisition devices were adjusted to record daily temperature and relative humidity from 9:00 a.m. to 5:00 a.m. before putting them in the solar tunnel drier. They were placed at the inlet of air for each drying zones until the drying process was completed. At the end of drying process, the data acquisition devices were plugged into the desktop and the recorded values were downloaded.

### 2.3 The drying process for open sun and solar drying

The drying experiment was performed between May 25, 2019 and May 30, 2019, when the days were fully sunny. Samples prepared were placed randomly in the three solar tunnel drying zones and in open sun. For the determination of drying characteristics, samples were placed on small wire mesh and put on each of the drying zones. Then, weight measurement was taken using a digital balance (ABJ220‐4M, WB1151070, Australia) in 30 min intervals starting from 9:00 a.m. to 5:00 p.m. Throughout the drying process, both relative humidity and temperature inside and outside the solar tunnel were recorded by using data acquisition devices (Testo, model 184 H1). Then, the drying process was stopped when the two consecutive measurements of the sample weights become constant (Taye, 2018).

### 2.4 Drying characteristics of taro slice

#### 2.4.1 Determination of moisture ratio

The moisture ratio (MR) of taro slice during drying experiments was calculated using Equation (1):

(1) $$\mathrm{MR}=\frac{M_{t}-M_{e}}{M_{o}-M_{e}}$$

where *M*_*t*_ is moisture content at any drying time, *M*_o_ is initial, and *M*_e_ is equilibrium moisture content.

The values of *M*_e_ are relatively little compared to those of *M*_*t*_ or *M*_o_, the error involved in the simplification is negligible (Goyal et al., 2008), and thus moisture ratio was calculated as Equation (2):

(2) $$\mathrm{MR}=\frac{M_{t}}{M_{o}}$$

#### 2.4.2 Determination of effective diffusivity (*D*_eff_) of taro slice

Doymaz (2010) methods were used to determine the mass diffusion of drying taro slices and it is shown in the equation 3:

(3) $$\frac{\partial M}{\partial t}=D_{\mathrm{eff}}{\text{∇}}^{2}\text{34𝑀}$$

where

The solution of diffusion equation (Equation 4) for slab geometry is solved by Crank (1975), and supposed uniform initial moisture distribution, negligible external resistance, constant diffusivity, and negligible shrinkage are as follows:

(4) $$\mathrm{MR}=\frac{8}{\pi^{2}}\sum_{n=0}^{\infty }\frac{1}{{\left(2n+1\right)}^{2}}\exp\left(\frac{{\left(2n+1\right)}^{2}{\mathrm{\pi D}}_{{\mathrm{eff}}^{t}}}{4L^{2}}\right)$$

where *D*_eff_ is the effective moisture diffusivity (m^2^/s), *n* is a positive integer, *t* is the drying time (s), and *L* is the half‐thickness of samples (m).

For long drying times, a limiting of Equation (5) is obtained and expressed in a logarithmic form:

(5) $$\ln\;\mathrm{MR}=\ln\left(\frac{8}{\pi^{2}}\right)-\left(\frac{\pi^{2}D_{{\mathrm{eff}}^{t}}}{4L^{2}}\right)$$

From Equation (5), a plot of ln MR versus drying time gave a straight line with a slope (*K*) of:

(6) $$\;K=\frac{\pi^{2}D_{\mathrm{eff}}}{4L^{2}}$$

#### 2.4.3 Models evaluation

To select the best model which expresses the drying behavior of taro slices, five different thin‐layer models were evaluated. To fit the experimental data, the models were evaluated based on statistical parameters, including the root mean square error (*RMSE*), reduced chi‐square (*χ* ^2^), coefficient of determination (*R* ^2^), and relative mean present error (*P*). The statistical parameters can be described in the following equations:

(7) $$\;R^{2}=1-\frac{\sum_{i=1}^{N}\;{\left({\mathrm{MR}}_{\mathrm{pre},i}-{\mathrm{MR}}_{\exp,i}\right)}^{2}}{\sum_{i=1}^{N}\;{\left({\mathrm{XMR}}_{\mathrm{pre},i}-{\mathrm{MR}}_{\exp,i}\right)}^{2}}$$

(8) $$\;X^{2}=\frac{\sum_{i}^{N}1{\left({\mathrm{MR}}_{\exp,i}-{\mathrm{MR}}_{\mathrm{pre}}\right)}^{2}\;}{N-n}$$

(9) $$\text{RMSE}={\left[\frac{1}{\;N\;}\sum_{i=1}^{N}\left({\mathrm{MR}}_{\mathrm{pre},i}-{\mathrm{MR}}_{\exp,i}\right)2\;\right]}^{\frac{1}{2}}$$

(10) $$P\%=\frac{100}{N}\sum \frac{\left|{\mathrm{MR}}_{\exp-{\mathrm{MR}}_{\mathrm{pre}}}\right|\;}{{\mathrm{MR}}_{\exp}}$$

where MR_pre_ is the predicted moisture ratio, MR_exp_ is the experimental moisture ratio, *N* is the number of observations, and *z* is the number of constants in the drying model (Sobukola et al., 2008).

#### 2.4.4 Specific heat capacity and thermal conductivity

The specific heat capacity and thermal conductivity were derived from proximate composition of the taro flour samples using the method of Barine and Victor (2016) (Equations 11 and 12), respectively.

(11) $$C_{p}=4.18X_{w}+0.908X_{a}+1.711X_{p}+1.929X_{f}+1.547X_{c}\ldots \ldots .$$

(12) $$K=0.58X_{w}+0.135X_{a}+0.155X_{p}+0.16X_{f}+0.25X_{c}$$

where *X*_w_ = mass fraction of water, *X*_a_ = mass fraction of ash, *X*_p_ = mass fraction of protein, *X*_f_ = mass fraction of fat, and *X*_c_ = mass fraction of carbohydrate.

### 2.5 Treatment combinations and experimental design

The experiment was laid out as a factorial combination of two main factors; namely drying zones with four levels [zone I, zone II, zone III, and open sun drying (OPD) were considered out of which OSD was considered as a control treatment]. Another factor of this experiment was slice thickness with three levels (2, 4, and 6 mm). The experiment was laid as a 4 × 3 factorial combination arranged in a randomized complete block design (RCBD) and replicated three times with 36 experimental units.

### 2.6 Data analysis

Reduced chi‐square (*χ* ^2^), the determination of coefficient (*R* ^2^), root mean square error (RMSE), and mean relative percent error (*P*) were used to evaluate the fitting quality of the data to the models. Their values were determined by nonlinear regression using MS, office Excel‐2016, and Minitab version 17 statistical software.

## 3 RESULTS AND DISCUSSION

### 3.1 Relative humidity and temperature of drying zones

The effects of air temperature and relative humidity are interrelated. Table 1 shows the relative humidity and temperature of various drying zones and the ambient air from May 25 to May 30, 2020 (during the sunny season). In comparison to the ambient, STD has a much greater temperature and lower relative humidity. As air flows from the dryer's inlet (zone 1) to its outlet (zone 3), STD's temperature rises. In the open sun, STD zone I was twice as hot as the surrounding air, whereas STD zone III was almost three times as hot. As the ambient air reached STD zone 3, the relative humidity of the air decreased. Since the higher temperature has a high water‐holding capacity, STD zone III has lower relative humidity.

**TABLE 1:** Temperature and relative humidity of the air in and out of STD.

Temperature and drying zones relative humidity		Average	Maximum	Minimum
Temperature (°C)	Open sun	22.8	28.7	19.2
	Zone I	44.9	53.3	35.7
	Zone II	51.7	62.5	38.0
	Zone III	62.3	70.0	49.5
Relative humidity (%)	Open sun	52.4	62.7	43.9
	Zone I	34.9	47.7	27.4
	Zone II	31.4	43.9	24.2
	Zone III	26.3	32.7	21.2

### 3.2 Effect of drying zones and slice thickness on drying characteristics of taro slice

#### 3.2.1 Effect of slice thickness on drying curve of taro slices

**FIGURE 2:**
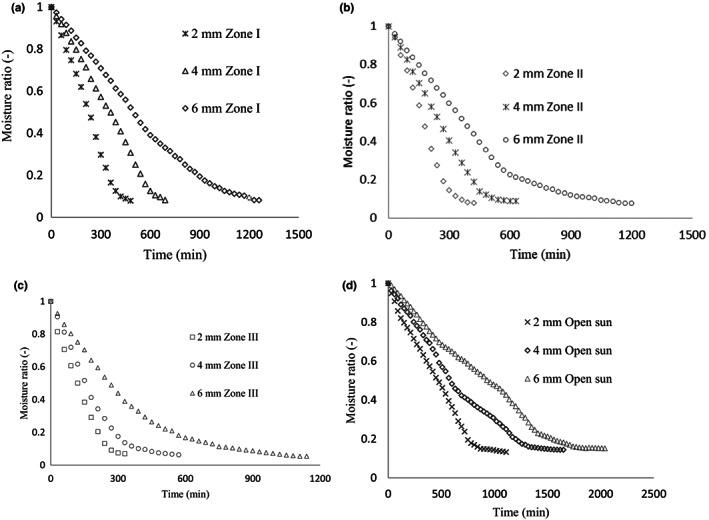
Drying curve of taro slice at constant drying zones within different slice thicknesses in the STD (a–c) and open sun (d) conditions.

Figure 2a–d depicts the influence of slice thickness on the drying curve of taro slices in the open sun and various drying zones. The taro slice dried in zone III with a drying thickness of 2 mm required the shortest period of time to dry (5.5 h), followed by a taro slice with a thickness of 4 mm (9.5 h), and finally, a taro slice with a thickness of 6 mm within the same zone (19 h). The longest drying hour (34 h) was spent drying the taro slice with a 6 mm thickness in the open sun. This is due to the fact that the higher temperature in drying zone 3 is responsible for the high removal of moisture, and the shortest distance in a thinner slice needs the shortest time to extract the moisture from the sample to the surface. This finding is agreed with the finding of Sanful et al. (2016), the less distance of thinly sliced samples required less time to extract water from the sample to the surface leading to faster drying. The finding of Doymaz (2012), who investigated the infrared drying of sweet potato slices, was in good agreement with the observation in this study. The increase in the drying time with increasing slice thickness was due to the effect that the exposed surface area resulted in an increased diffusion pathway of moisture out of the sweet potato slices (Doymaz, 2012).

#### 3.2.2 Effect of drying zones on drying curve of taro slice

**FIGURE 3:**
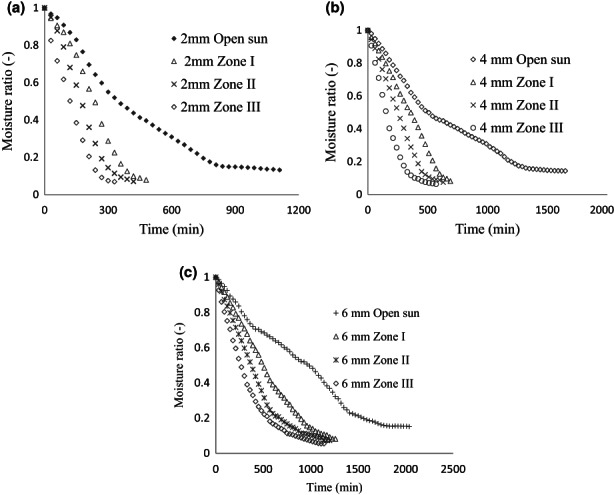
Drying curve of taro slice at constant slice thickness within different drying zones (a–c).

Figure 3a–c shows the effect of drying zones on moisture removal of taro slices at constant slice thickness. Different drying zones remove taro slices' moisture at varying rates despite their constant thickness. Among the drying zones under zone III, the moisture removal was high and a shorter time is taken to reach the equilibrium moisture content. Compared to the samples dried in zones I and II, zone III had a higher temperature (62.3°C) and lower relative humidity (26.3%). Taro slices dried in the open sun drying took the longest time as compared to the samples having the same thickness dried in STD. The presence of higher the temperature of the air in STD zone III compared to the remaining zones results in a higher temperature difference between the samples and air. Which is the driving force for heat and mass transfer between the air and the samples in the zone III. The sample produced latent heat to evaporate water from its surface after absorbing heat from the drying air (Taye, 2018). An increase in the drying temperature results in an increase in moisture removal and a decrease in the drying times (Gupta & Patil, 2014). Therefore, drying zone temperature had an effect on the drying time and moisture removal as reported in a previous study (Khazaei et al., 2008).

#### 3.2.3 Evaluation of the drying models

The drying data for different drying zones fit to the selected five thin‐layer drying models are presented in Table 2. To evaluate the parameters of the models, nonlinear regression analysis was used. The best model expressing the thin‐layer drying behavior of taro slices was chosen as the one with the lowest value of RMSE, *χ* ^2^, and *P*, and the highest value *R* ^2^ (Table 2).

**TABLE 2:** Fitting of the models for zones I, II, and III and open sun.

Slice thickness	Models	RMSE	*X* ^2^	*R* ^2^	*P* (%)
Zone I					
2 mm	Lewis	0.00610	0.00078	.94196	21.9
	Henderson and Pabis	0.00246	0.00127	.99172	12.8
	Logarithmic	0.00139	0.00013	.99396	5.54
	Page	0.00370	0.00020	.98751	13.3
	Wang and Singha	0.00284	0.00110	.99179	9.58
4 mm	Lewis	0.01357	0.00116	.93136	24.9
	Henderson and Pabis	0.00502	0.00144	.97874	15.9
	Logarithmic	0.00383	0.00035	.98681	6.91
	Page	0.00324	0.00116	.98336	17.2
	Wang and Singha	0.02216	0.00050	.98413	11.8
6 mm	Lewis	0.00477	0.01046	.98228	19.7
	Henderson and Pabis	0.08490	0.00759	.86340	25.3
	Logarithmic	0.00373	0.00417	.98732	7.77
	Page	0.00047	0.00943	.98463	14.4
	Wang and Singha	0.00340	0.00889	.98524	12.7
Zone II					
2 mm	Lewis	0.00906	0.00095	.95768	18.9
	Henderson and Pabis	0.00316	0.01498	.98849	16.2
	Logarithmic	0.00266	0.00026	.99032	7.22
	Page	0.00320	0.00150	.98835	14.4
	Wang and Singha	0.00315	0.00106	.99422	11.1
4 mm	Lewis	0.00502	0.00168	.94306	25.6
	Henderson and Pabis	0.00105	0.00067	.99604	13.8
	Logarithmic	0.00104	0.00010	.99606	5.82
	Page	0.00255	0.00118	.96536	9.15
	Wang and Singha	0.00242	0.00042	.99840	8.04
6 mm	Lewis	0.00239	0.00259	.93753	28.5
	Henderson and Pabis	0.00479	0.00106	.97350	19.3
	Logarithmic	0.00417	0.00041	.98700	9.33
	Page	0.00607	0.001202	.96627	22.8
	Wang and Singha	0.00606	0.00120	.96631	21.6
Zone III					
2 mm	Lewis	0.00140	0.00049	.93345	28.1
	Henderson and Pabis	0.00415	0.00178	.98777	11.4
	Logarithmic	0.00338	0.00338	.99705	6.39
	Page	0.00408	0.01765	.98798	14.5
	Wang and Singha	0.00832	0.00079	.99057	9.68
4 mm	Lewis	0.00159	0.00041	.95571	19.9
	Henderson and Pabis	0.00519	0.00157	.97624	17.5
	Logarithmic	0.00343	0.0003	.99362	3.91
	Page	0.00257	0.00110	.98832	14.2
	Wang and Singha	0.00380	0.00081	.99353	10.45
6 mm	Lewis	0.00178	0.03175	.94550	25.0
	Henderson and Pabis	0.00373	0.00978	.98013	15.2
	Logarithmic	0.00124	0.00274	.99044	7.41
	Page	0.00201	0.00718	.98937	10.57
	Wang and Singha	0.00275	0.00671	.98572	12.5
Open sun					
2 mm	Lewis	0.00408	0.00097	.93443	27.1
	Henderson and Pabis	0.00174	0.00667	.99195	12.4
	Logarithmic	0.00165	0.00012	.99236	8.58
	Page	0.00408	0.00176	.98798	15.0
	Wang and Singha	0.00237	0.00078	.98899	13.9
4 mm	Lewis	0.00239	0.01063	.95997	23.3
	Henderson and Pabis	0.00128	0.00475	.99366	7.33
	Logarithmic	0.01494	0.00018	.99537	7.07
	Page	0.00257	0.00110	.98832	12.2
	Wang and Singha	0.00278	0.00069	.98624	11.4
6 mm	Lewis	0.01357	0.00629	.95888	23.3
	Henderson and Pabis	0.00427	0.00078	.98501	11.5
	Logarithmic	0.00332	0.00032	.98835	9.89
	Page	0.01706	0.01572	.93753	28.3
	Wang and Singha	0.00155	0.00047	.98459	10.35

When compared to the other four models used in this study, the logarithmic model was determined to be the most appropriate model for explaining the drying processes of taro slices dried in different drying zones I, II, and III and open sun. Since the lowest value of *χ* ^2^, RMSE, and *P* and the highest value of *R* ^2^ were obtained for logarithmic model compared to the remaining models, the result of this study was in agreement with a previous finding for peach (Zhu & Shen, 2014).

#### 3.2.4 Effective moisture diffusivity (*D*_eff_)

The effect of drying zones and thickness of slices on moisture diffusivity of taro slices is presented in Table 3. The *D*_eff_ values for taro slices ranged from 1.19 × 10^10^ to 6.57 × 10^−09^ m^2^/s. The highest moisture diffusivity (6.57 × 10^−09^ m^2^/s) was observed for the thinner slices (2 mm) dried at hotter drying zone (zone III), whereas the lowest moisture diffusivity (1.19 × 10^−10^) was obtained for the thicker slices (6 mm) dried in the open sun. The *D*_eff_ increased with decreasing slice thickness increasing in air temperature and decreasing slice thickness of the samples (Babalis & Belessiotis, 2004; Titilope et al., 2020). The average value of the *D*_eff_ for agricultural products is in the range 10^−9^ to 10^−11^ m^2^/s. This is in agreement with the finding of different authors (Babalis & Belessiotis, 2004). Babalis & Belessiotis, 2004, found that the *D*_eff_ increases with a decrease in the slice thickness of the fig sample and an increase in the air temperature.

**TABLE 3:** Effective moisture diffusivity for different zones and various slice thicknesses of dried taro slices.

Drying zones	Slice thickness (mm)	Diffusivity (m^2^/s)
Zone I	2	2.83 × 10^−09^
	4	2.59 × 10^−09^
	6	2.39 × 10^−09^
Zone II	2	6.35 × 10^−09^
	4	5.84 × 10^−09^
	6	5.11 × 10^−09^
Zone III	2	6.57 × 10^−09^
	4	5.93 × 10^−09^
	6	5.32 × 10^−09^
Open sun	2	1.78 × 10^−09^
	4	1.51 × 10^−10^
	6	1.19 × 10^−10^

#### 3.2.5 Specific heat capacity (*C*_p_) and thermal conductivity (*k*) of taro flour

Tables 4 and 5 show the impact of the drying zones and thickness on the specific heat capacity and thermal conductivity of taro flour, respectively. The *C*_p_ of taro flour ranged between 1.760 and 1.799 kJ/kg °C. It was significantly affected by drying zones but not by thickness and the interaction effect of thickness and drying zones. The *C*_p_ value of taro flour decreased as the drying zone approached the exit (zone 3) or as the temperature of the air increased, which is in agreement with Ajala et al. (2014). They reported that the *C*_p_ value of cocoyam flour decreased as the air's drying temperature rose. The energy required for processing taro flour prepared by drying in open sun and drying zone 1 were 1.799 and 1.794 kJ/kg of the sample, respectively, which is significantly higher than the energy required for processing samples prepared from zone 2 (1.776 kJ/kg) and zone 3 (1.760 kJ/kg). This result agreed with the report in a previous study for anchote flour; *C*_p_: between 1.71 and 1.78 kJ/kg °C (Bikila et al., 2020).

**TABLE 4:** Mean values for specific heat capacity and thermal conductivity of taro obtained from different drying zones.

Zone	*C*_p_ (kJ/kg °C)	*K* (W/m °C)
1	1.79902^a^ ± 0.004	0.270380^b^ ± 0.0006
2	1.77643^b^ ± 0.003	0.268106^c^ ± 0.0005
3	1.76066^c^ ± 0.009	0.267721^c^ ± 0.0009
Open sun	1.79456^a^ ± 0.003	0.273392^a^ ± 0.0008
LCD	0.0054	0.0007

*Note*: Means that do not share a letter are significantly different.

**TABLE 5:** Mean values for specific heat capacity and thermal conductivity of taro obtained from different thickness.

Thickness (mm)	*C*_p_ (kJ/kg °C)	*K* (W/m °C)
2	1.779^a^ ± 0.016	0.269^a^ ± 0.002
4	1.783^a^ ± 0.014	0.270^a^ ± 0.018
6	1.786^a^ ± 0.016	0.271^a^ ± 0.002
LCD	0.015	0.0023

*Note*: Means that do not share a letter are significantly different.

Similar to *C*_p_, *K* of taro flour was strongly impacted by drying zones but not by thickness or the effect of thickness and drying zones combination. The taro flour *k* ranged between 0.267 and 0.273 W/m °C. Taro flour that had been dried in the open sun had a higher *K* value (0.273 W/m °C), whereas the lowest value of *K* of taro flour (0.271 W/m °C) was recorded for the sample that had been dried in drying zone 3. The decrease in *K* of taro flour is due to an inverse relationship of *K* with drying temperature similar to the report of Ajala et al. (2014). This result agreed with the report of Bikila et al. (2020). They found that the *K* of anchote flour ranged between 0.260 and 0.275 W/m °C.

## 4 CONCLUSIONS

In this study, solar tunnel drying zones and slice thickness were used to investigate their effect on the thermal properties and drying characteristics of taro slices. Based on the results, the logarithmic model gave the best representation of drying data under all experimental conditions. Thinner slice samples dried around the exit of the dryer (drying zone 3) took the shortest time to reach the required moisture level. *D*_eff_, f *C*_p_ and *K* c In comparison to the samples that had been dried in other drying zones and control, the thinest slices that had been dried in drying zone 3 had the highest value of Deff, lowest value of Cp and K. Results showed that slices of taro can be dried faster and with better quality by using a solar tunnel drier rather than a conventional dryer. It could be concluded that slice thickness and drier zones have a significant impact on the drying behavior and thermal properties of taro slices.

## FUNDING INFORMATION

This research did not receive any specific grant for publication from funding agencies in the public, commercial, or not‐for‐profit sectors.

## CONFLICT OF INTEREST

The authors declare no conflict of interest.

## ETHICAL APPROVAL

This study does not involve any animal or human testing.

## Data Availability

The data used to report these results are available from the corresponding author upon request.
